# Exploring Psychosocial Risk Factors Among Spanish Nurses: Links to Health and Professional Variables

**DOI:** 10.1155/jonm/5531311

**Published:** 2025-08-26

**Authors:** Ángela Narbona-Gálvez, Juan Gómez-Salgado, Regina Allande-Cussó, Carlos Ruiz-Frutos, Diego Ayuso-Murillo, María Guadalupe Fontán-Vinagre, Blanca Prieto-Callejero, Krzysztof Goniewicz, Javier Fagundo-Rivera, Antonio Castaño-Seiquer

**Affiliations:** ^1^School of Doctorate, University of Huelva, Huelva, Spain; ^2^Department of Sociology, Social Work and Public Health, Faculty of Labour Sciences, University of Huelva, Huelva, Spain; ^3^Safety and Health Postgraduate Programme, Universidad Espíritu Santo, Guayaquil, Ecuador; ^4^Department of Nursing, Faculty of Nursing, Physiotherapy and Podiatry. University of Seville, Sevilla, Spain; ^5^General Secretariat, General Nursing Council of Spain, Madrid, Spain; ^6^Spanish Institute for Nursing Research, General Nursing Council of Spain, Madrid, Spain; ^7^Department of Nursing, University of Huelva, Huelva, Spain; ^8^Department of Security, Polish Air Force University, Deblin, Poland; ^9^Red Cross University Nursing Centre, University of Seville, Seville, Spain; ^10^Department of Stomatology, Faculty of Odontology, University of Seville, Seville, Spain

**Keywords:** health perception, healthcare services, nursing, occupational stress, occupational well-being, psychosocial risks

## Abstract

**Objective:** To identify factors associated with the perception of psychosocial risks among practising nurses in Spain and to examine their relationship with personal, professional and health-related characteristics.

**Design:** Observational, cross-sectional and correlational study.

**Participants:** A total of 2765 nurses completed an online questionnaire between March and June 2023. The survey was distributed via professional networks and the General Nursing Council of Spain.

**Methods:** Descriptive and bivariate analyses were conducted, along with a categorical regression to identify factors associated with perceived psychosocial risk (measured using the ISTAS_Enfermería scale). Model assumptions were tested and multicollinearity was assessed. The model included self-reported sociodemographic, occupational and mental health variables.

**Results:** Female sex, being under 41 years of age, working night or rotating shifts, recent use of psychotropic medication and symptoms of anxiety or depression were all associated with higher levels of perceived psychosocial risks. These variables explained 29.6% of the variance. Due to the cross-sectional design, no causal relationships can be inferred.

**Conclusions:** Certain professional profiles appear to be more vulnerable to perceived psychosocial risks that are associated with their personal, occupational and health characteristics. These associations are likely to be bidirectional and context-dependent. Longitudinal research is needed to better understand causal mechanisms and to guide personalised preventive interventions.

**Summary**

• What is already known
◦ Psychosocial risks, such as work overload or lack of support, are well-established determinants of psychological distress, burnout, and the intention to leave the nursing profession.
◦ Health conditions such as chronic illnesses, poor self-perceived health and high stress levels have previously been linked to worse health outcomes and workplace issues among healthcare professionals.
◦ Younger age and job insecurity are often associated with greater stress and vulnerability among nurses.
• What this paper adds
◦ Confirms previously reported links between psychosocial risks and health-related factors—such as chronic illness, self-perceived health and stress—while providing contextualised evidence from a large national sample of Spanish nurses.
◦ Highlights that younger age, nonmarital cohabitation and holding a Master's degree are associated with a higher perception of psychosocial risks in the Spanish nursing context. These associations may reflect not only actual exposure but also greater critical awareness or the influence of unmeasured occupational factors such as job stability or workload.
◦ Suggests a potential link between regular psychotropic drug use, sick leave and perceived psychosocial risks, raising questions about the role of coping mechanisms in shaping risk perception.

## 1. Introduction

The well-being of workers, which encompasses both physical and psychological health, is a key factor that affects the productivity and success of any organisation [1]. In healthcare, and specifically in nursing, occupational health becomes particularly relevant due to the emotional and physical demands of the job. The 2010 ‘Towards Better Work and Well-being' conference emphasised the need for a holistic approach to occupational health, highlighting the importance of addressing the effects of an ageing workforce and the impact of chronic illnesses on the healthcare staff [2]. In this context, psychosocial risks, such as work overload, role conflict, lack of organisational support and continuous exposure to patient suffering, have been identified as determinants of psychological distress, emotional exhaustion and intention to leave the profession among the nursing staff [3]. In the context of this study, ‘psychosocial risks' are operationally defined as the subjective perception of work environment conditions that may affect the psychological and physical health of nursing professionals. This perception is specifically measured through the dimensions assessed by the ISTAS_Enfermería questionnaire, which addresses aspects such as work stress, job satisfaction, well-being at work, job insecurity and dual presence. Recent studies in the Spanish context highlight that these risks are directly associated with symptoms of anxiety, depression and burnout, thus compromising both the health of professionals and the quality of care [4].

In recent years, psychosocial risks in the nursing sector have intensified due to various factors, with the COVID-19 pandemic being one of the main catalysts for this phenomenon [5]. This global health crisis led to a drastic increase in workload and extreme psychological pressure, which exacerbated pre-existing mental health problems [6]. A recent meta-analysis found that the prevalence of anxiety among healthcare workers during the pandemic was at 40%, depression at 37%, distress at 37% and post-traumatic stress disorder at 49% [6].

In addition to the pandemic, other structural factors contribute to the deterioration of the mental health of nurses. Factors such as work overload, long shifts and the introduction of new technologies have increased work pressure, which translates into increased stress, absenteeism and a decrease in the quality of care provided [7].

Also, factors such as high workload, lack of support and strained working relationships increase the risk of anxiety, depression and burnout among nurses. These conditions not only affect their well-being but also diminish the quality of care they provide to patients [8].

Globally, the shortage of nurses and an ageing workforce exacerbate these problems, underscoring an urgent need to improve working conditions and promote a more supportive work environment [9]. A positive work environment can reduce stress and improve patient safety, but newly graduated nurses face challenges related to the gap between theory and practice, which increases stress and the risk of errors in care [10].

Despite the extensive documentation of psychosocial risks in the nursing field provided by the international literature, there is a specific gap in research that comprehensively addresses the interrelationship between these risks and various sociodemographic and health variables among Spanish nursing staff. Recent studies have assessed psychosocial risks in Spanish nurses, finding significant differences according to sociodemographic and employment variables [11]. However, few have explored in depth the association of factors such as the presence of chronic diseases, self-perceived health, stress, history of sick leave and the use of psychotropic drugs with the perception of psychosocial risks in this specific population. The feminised nature of the nursing profession in Spain (with 87.3% of women in this study sample) underlines the importance of understanding differences by sex regarding psychosocial risks and their impact on the health of female workers. This is especially important in the context of an ageing nursing workforce, where women may experience greater exposure to these risks and greater difficulties in balancing work and family life [12]. The paucity of longitudinal studies in Spain that track the evolution of these risks and their long-term consequences also represents a significant limitation that this preliminary study seeks to partially address by providing a solid foundation for future research.

In this study, the term ‘ageing of the workforce' is employed to denote the specific phenomenon of the ageing demographic of nursing staff in Spain. General demographic ageing significantly impacts the nursing workforce, leading to an increase in the average age of active professionals and a decline in the replacement rate for retirees [13]. This phenomenon is of critical importance to the Spanish healthcare system, as an ageing nursing workforce may experience an increase in the prevalence of chronic diseases, greater vulnerability to occupational stress and, potentially, a higher number of sick leave days [14]. Therefore, it is crucial to investigate how these demographic changes within the nursing profession directly affect the perception of psychosocial risks and the health of these professionals. It is important to note that, while studies suggest that older workers may have more positive well-being outcomes [12], younger nurses may experience higher levels of stress due to lack of experience and job instability, highlighting the complexity of the age variable in this context [11].

In addition, job insecurity also has a negative impact on nurses, especially on those who are younger. This contributes to the abandonment of the profession and negatively affects the physical and mental health of nurses [9].

Besides psychosocial risks, other health-related variables that affect nurses must also be considered, such as the presence of chronic illnesses. Several studies have shown a high prevalence of chronic pathologies among the nursing staff, especially those of a musculoskeletal (such as low back pain) or cardiovascular nature, which are directly related to the physical and postural characteristics of the work, and to rotating shifts and chronic stress [16]. These conditions not only impact the professional's quality of life but also their performance and retention in the work environment.

Another variable of great interest is self-perceived health status, which acts as a subjective indicator of the physical and emotional state of workers. A low perception of health has been linked to high levels of fatigue, stress, low work engagement and a greater intention to leave the profession [17]. This perception also correlates with a higher number of sick leaves, which in the health sector not only puts a strain on the rest of the working team but also represents a significant cost to the health system [18].

In this context, the use of psychoactive substances can be understood as a behaviour associated with exposure to psychosocial risks, rather than as a risk in itself. Various studies have identified nursing staff as a group vulnerable to the use of substances such as alcohol, tobacco, benzodiazepines or even prescription drugs (antidepressants and opioid analgesics), often used as a coping strategy for stress or work-related burnout [19]. Despite the ongoing taboo surrounding the subject in healthcare settings, the available evidence suggests that a considerable proportion of professionals use these substances. This practice not only poses a threat to their personal health but also jeopardises the quality and safety of the care they provide [20]. In this study, the consumption of alcohol, tobacco and other substances was included as a predictive variable in the statistical model, and it is evaluated using self-reported items that collected data on the regular consumption of these substances in the previous year. This information enables an assessment of its potential correlation with the perception of psychosocial risks. However, caution should be given to the interpretation of the findings, due to the risk of underreporting and the causal complexity of these behaviours.

Drawing upon these findings, the present study aims to contribute to the available literature by conducting a comprehensive analysis of the prevalence of varying degrees of perceived psychosocial risks among Spanish nursing staff. Specifically, the objective is to ascertain potential disparities in the perception of psychosocial risk according to key sociodemographic variables, including sex, age, marital status and educational level. Additionally, a primary objective is to determine the association between the perception of psychosocial risks and various health-related variables, including the presence of chronic diseases, self-perceived health status, stress levels, history of sick leave and the use of psychoactive substances among Spanish nurses. Ultimately, the objective of the present study is to identify the most significant sociodemographic and health factors in predicting the perception of psychosocial risks within the context of Spanish nursing.

In light of the relevance of these factors, this study adopts an exploratory approach to examine and analyse psychosocial risks in Spanish nursing and their relationship with certain health-related variables, with the aim of identifying key associations and potential predictors. Understanding these dynamics will support the development of strategies to improve the working conditions and well-being of nursing professionals, thereby enhancing both their quality of life and the care they provide to patients.

## 2. Methods

### 2.1. Design

A cross-sectional descriptive study based on questionnaires.

### 2.2. Population and Sample

The present study relied on a population of a total of 345,969 registered nurses in Spain, according to data corresponding to the year 2023. The research had a national scope, covering registered nurses in all Spanish autonomous regions, and considered the participation of nurses who met the following inclusion criteria: (i) performing the healthcare functions of the profession, (ii) residing in Spain and (iii) actively practising the profession at the time of the study. Nurses who did not meet these criteria were excluded, as well as the following: (i) professionals with degrees validated abroad who had not yet begun to practice in Spain, (ii) nurses who performed noncare functions and (iii) those who worked outside the country.

The data were collected from nurses working in both primary and specialised care settings, either within the National Health Service (NHS) or in private institutions such as private clinics, nursing homes for the elderly, among others.

To determine the sample size, a calculation was made considering a margin of error of 5%, a confidence level of 95% and a heterogeneity of 50%, which determined a minimum requirement of 384 participants. However, the final number of nurses in the sample was 2,765, significantly exceeding the initial estimate and, consequently, increasing the statistical power of the study. Based on the final sample size of 2765 and assuming a medium effect size (*f*^2^ = 0.15), the statistical power of the multivariate model was estimated to be above 0.99 (*α* = 0.05), which exceeds the conventional threshold of 0.80. Similar levels of power were achieved for the bivariate analyses, even in the case of small effect sizes.

The sample selection was carried out by means of nonprobabilistic or ‘snowball' sampling, starting with nursing professionals who agreed to participate and who, in turn, facilitated the incorporation of new participants through dissemination. Participation in the study was completely voluntary, and the confidentiality of the information provided was guaranteed. Prior to data collection, all participants gave informed consent, ensuring compliance with the ethical principles of the study.

### 2.3. Variables

The present study examines sociodemographic, psychosocial and health-related variables. To assess the sociodemographic profile, questions on sex, age, marital status and level of education attained were included. The full list of response categories for each variable is presented in Table 1. Regarding the level of education, the questionnaire also collected data on training and professional development. ‘Expertise' referred to the participant's self-reported level of clinical specialisation or postgraduate training within the nursing field (e.g., wound care and critical care). ‘Vocational training' referred to previous technical or professional education obtained prior to or alongside university-level nursing studies.

Health-related variables included questions on the presence of chronic illnesses, health status, level of stress, whether they were on sick leave, and if so, the type of sick leave, and how often they used different substances: alcohol, tobacco, cannabis, heroin, amphetamines, benzodiazepines and antidepressants.

The level of psychosocial risks was assessed using the ISTAS_Enfermería scale. This tool is a validated adaptation for the nursing population, designed to measure specific psychosocial factors that affect the performance and well-being of these professionals in the workplace. In this study, the term ‘psychosocial risk' is operationally quantified through the scores obtained in the five dimensions of the scale: ‘Support at work', ‘Job satisfaction', ‘Well-being at work', ‘Job insecurity', and ‘Double presence'. The total score, which ranges from 0 (*most favourable*) to 4 (*least favourable*), represents the overall level of perception of psychosocial risk, with higher scores indicating a greater perception of risk. The tool measures psychosocial factors affecting the work performance of nurses, such as work stress and quality of life at work. The validation of the tool in the nursing population obtained a Cronbach's alpha value of 0.7. The scale presents a factorial structure of 5 dimensions with 15 items, which explain 63.3% of the variance, and shows an optimal fit in the parameters calculated by confirmatory factor analysis (values higher than 0.90 for the TLI, NFI and CFI, lower than 0.08 for the SRMR and lower than 0.08 for the RMSEA) [11]. The reliability and CFA fit indices cited refer to the original validation study by Narbona-Gálvez et al. [11], which reported satisfactory psychometric properties of the ISTAS_Enfermería scale. No additional CFA was conducted in the present study.

The ISTAS_Enfermería instrument [11] was selected for this study given its specific adaptation to the nursing profession, and it is derived from the Spanish version of the Copenhagen Psychosocial Questionnaire (COPSOQ) [21]. Unlike other generic instruments used in occupational health (such as the Job Content Questionnaire or the effort–reward imbalance [ERI] model), ISTAS_Enfermería focuses on dimensions that are particularly relevant in the nursing context, such as emotional demands, job satisfaction and dual presence. Its structure and language are tailored to the reality of clinical nursing work in Spain, which enhances its validity and applicability to this professional group [11].

This tool comprises five dimensions: (1) *psychological demands* (5 items), (2) *active work and development possibilities* (4 items), (3) *social support and quality of leadership* (4 items), (4) *compensations* (3 items) and (5) d*ouble presence* (2 items). Each item is scored on a Likert scale, and dimension scores are standardised from 0 to 20 to allow comparability. The items address aspects such as mental workload, autonomy, perceived support, salary/benefits and work–life balance. The scores varying according to the dimension: in ‘Support at work' and ‘Job satisfaction', items are rated from 0 (*Always*) to 4 (*Never*), while in ‘Well-being at work', ‘Job insecurity' and ‘Double presence', items are rated from 4 (*Always*) to 0 (*Never*). The total score is obtained by adding the values of all items, ranging from 0 (*most favourable*) to 60 (*least favourable*) (Supporting Table 1).

### 2.4. Procedure

The data collection process took place over a period of 3 months, from 1 August to 31 October 2023. For this purpose, a digital questionnaire was elaborated using the Google Forms © platform, which was disseminated through social networks and professional nursing groups. This questionnaire included questions related to the different variables to be studied. As a prerequisite for access, participants had to confirm their status as practising nurses and accept the informed consent. Participation was voluntary, and responses were collected through an anonymous online questionnaire, with no personal identifiers stored or linked to the data. Therefore, to minimise the risk of duplicate responses or inclusion of individuals outside the target population, the questionnaire included a mandatory screening item at the beginning, requiring participants to confirm that they were currently practising nurses in Spain. Responses were only accepted if this criterion was explicitly marked. In addition, the research team monitored the dataset to identify potential duplicate entries (e.g., repeated timestamps or identical demographic patterns), but no significant anomalies were detected. Although participation was anonymous and no IP filtering was used to ensure accessibility, data quality was reviewed during preprocessing to confirm internal consistency. The estimated time to complete the questionnaire was approximately 7–10 min.

The research team managed the distribution of the questionnaire and ensured its dissemination in channels intended for nursing professionals. To broaden its scope, the study was supported by the General Council of Nursing of Spain, which actively collaborated in its dissemination. Specifically, the questionnaire was shared through targeted newsletters and emails sent to professional mailing lists of registered nurses interested in participating in research studies across the 17 autonomous regions in Spain.

### 2.5. Data Analysis

Both univariate and bivariate descriptive analyses were carried out using SPSS Statistics © v26 software [22]. To determine whether the data followed a normal distribution, the Kolmogorov–Smirnov test was applied, with results showing a value of *p* < 0.05, indicating that the distribution was not normal. For this reason, nonparametric statistical tests were used, such as the Mann–Whitney U and the Kruskal–Wallis tests for comparisons between groups, as well as Kendall's Tau B coefficient to assess correlations between variables.

A categorical regression (CATREG) model was conducted using the overall score of perceived psychosocial risks (total ISTAS_Enfermería score) as the dependent variable. The independent variables were sex, age, marital status, education level, presence of chronic illness, self-perceived health status, use of psychotropic medication and history of sick leave. The CATREG analysis was applied, which is suitable for variables of a qualitative nature [24]. This method included components characteristic of classical regression, such as the coefficient of determination (*R*^2^), the analysis of variance associated with the regression, and the assessment of the significance of the model parameters. The selection of variables for the multivariate model was based on both theoretical relevance, as supported by the literature, and results from the bivariate analyses. In order to guarantee a correct adaptation of the variables to the model, the optimal scaling function available in SPSS© was used [22].

### 2.6. Ethical Aspects

This study was approved by the Provincial Research Ethics Committee of Huelva (Code 1520-N-23) and was conducted in accordance with the ethical principles set out in the Declaration of Helsinki [26]. All participants gave their informed consent after reviewing the study information and confirming their current professional status as nurses. The questionnaire was anonymous and self-administered, and access to participation required explicit confirmation of consent.

The collection, processing and storage of data complied with the applicable data protection legislation, including Regulation (EU) 2016/679 of the European Parliament and of the Council on the protection of personal data (GDPR) [23], and Spain's Organic Law 3/2018 on the Protection of Personal Data and Guarantee of Digital Rights (LOPDGDD). Appropriate technical and organisational measures were implemented to guarantee data confidentiality, and all information was stored in a secure database, accessible only to the research team.

## 3. Results

### 3.1. Descriptive Analysis

The research involved 2765 nursing professionals from various regions of Spain. The average age of respondents was approximately 41 years, with a predominantly female sample (87.3%), and a very small proportion identified themselves as nonbinary (*n* = 13; 0.5%). The mean score on the ISTAS21 questionnaire was 1.78 (SD = 0.503), with no statistically significant differences by sex (*p*=0.503). The participants' ages ranged from 21 to 65 years, with a median age of 42.1 (IQR: 34–51). Age groups were distributed as follows: under 30 years (18.5%), 30–39 years (27.3%), 40–49 year (29.6%); and 50 years or older (24.6%). The main results of the descriptive bivariate analysis, relating to sociodemographic and occupational variables, are presented in Tables 1 and 2.

In addition to the global ISTAS_Enfermería score, the mean values of the five psychosocial risk dimensions were analysed. The highest scores were observed in *psychological demands* ($\overset{¯}{x}$ = 12.32; SD = 2.82), followed by *active work and development possibilities* ($\overset{¯}{x}$ = 8.75; SD = 3.06) and *social support and quality of leadership* ($\overset{¯}{x}$ = 8.42; SD = 3.23). Lower scores were found in *compensations* ($\overset{¯}{x}$ = 5.73; SD = 2.91) and *double presence* ($\overset{¯}{x}$ = 3.67; SD = 1.74). These results indicate that perceived psychological overload and demands are the most salient risks, while work–life balance (double presence) and compensation also represent significant areas of concern. Bivariate analyses were conducted to explore the association between sociodemographic variables and each psychosocial risk dimension. Spearman's rho revealed statistically significant but weak negative correlations between age and most risk dimensions, particularly with *compensations* (Tau-b = −0.34; *p* < 0.001), *social support* (Tau-b = −0.10; *p* < 0.001) and *active work* (Tau-b = −0.07; *p* < 0.001). No significant association was found between age and *double presence* (Tau-b = 0.01; *p*=0.523). In addition, Kruskal–Wallis tests showed significant differences in *psychological demands* (*H* = 18.32; *p*=0.005), *social support* (*H* = 23.96; *p* < 0.001), *compensations* (*H* = 110.85; *p* < 0.001) and *double presence* (*H* = 96.18; *p* < 0.001) regarding marital status and educational level. These findings indicate that certain sociodemographic factors may influence the perception of specific psychosocial risks in the nursing workforce.

The findings suggest statistically significant differences in ISTAS_Enfermería scores according to marital status and level of education attained. The Kruskal–Wallis test revealed significant differences in psychosocial risk scores related to marital status and education level. Specifically, participants with a Master's degree showed higher perceived risk scores compared to those with either doctoral or undergraduate degrees. Similarly, married individuals scored lower than those who were single or separated. A negative and significant correlation was also identified between age and the scores (Tau-b = −0.12, *p* < 0.001), suggesting that the perception of psychosocial risks tends to decrease with increasing age. In contrast, no significant differences were observed according to sex (*p*=0.503), despite women constituting the majority of the sample (87.3%).

The mean ISTAS_Enfermería scores showed statistically significant differences according to the presence of chronic illness, self-perceived health status, level of stress, sick leaves and use of different toxic substances (*p*=0.001). Higher values were observed in professionals with chronic illness ($\overset{¯}{x}$ = 1.841), in those who rated their health as poor ($\overset{¯}{x}$ = 2.292) or fair ($\overset{¯}{x}$ = 2.064), and in those who had had two or more sick leaves in the previous year ($\overset{¯}{x}$ = 2.027). In addition, a positive correlation was found between perceived stress and ISTAS_Enfermería scores (Tau-b = 0.318, *p*=0.001).

Concerning the use of psychotropic drugs, higher scores were found among regular users of benzodiazepines ($\overset{¯}{x}$ = 2.031) and regular users of antidepressants ($\overset{¯}{x}$ = 2.095). No significant differences were found by the type of sick leave or regarding the use of alcohol, tobacco, cannabis, heroin or amphetamines.

### 3.2. Multivariate Regression Analysis

A CATREG analysis was carried out using the total score of the ISTAS_Enfermería scale as the dependent variable and those variables that showed significant differences in the bivariate analysis as predictors (see Tables 1 and 2). To indicate the precision of the associations, model estimates were reported with their corresponding 95% confidence intervals. The results of the regression analysis are shown below (Table 3).

The regression model applied is significant and explains 29.6% of the variance of the data.

Accordingly, nurses who were cohabiting with a partner ($\overset{¯}{x}$ = 1.840) had a 0.072 times higher perception of psychosocial risks than those who were married ($\overset{¯}{x}$ = 1.722), single ($\overset{¯}{x}$ = 1.819), separated ($\overset{¯}{x}$ = 1.824) or widowed ($\overset{¯}{x}$ = 1.814). This suggests that marital status significantly influences the perception of the work environment, with the condition ‘cohabiting without being married' being associated with a higher perception of risks.

In terms of educational level, nurses with a Master's degree ($\overset{¯}{x}$ = 1.831) or a Bachelor's degree (new model) ($\overset{¯}{x}$ = 1.809) presented a perception of psychosocial risks 0.104 times higher than those with Lower University Studies (former model) ($\overset{¯}{x}$ = 1.722) or a Doctorate ($\overset{¯}{x}$ = 1.714). This shows a significant relationship between the educational level and the perception of the work environment: the more technical or academic the level, the lower the perception of risk.

Regarding sick leaves, nurses who had had one sick leave ($\overset{¯}{x}$ = 1.842) or two or more sick leaves ($\overset{¯}{x}$ = 2.027) in the previous year had a higher perception of risks than those who had not had any ($\overset{¯}{x}$ = 1.689). However, the model shows a negative association (*β* = −0.135; *p* < 0.001), indicating that, overall, those who had had sick leaves had a lower perception psychosocial risks, i.e., 0.135 times lower ($\overset{¯}{x}$ = 1.689) than those who had not had any sick leave.

With respect to benzodiazepine use, nurses who used benzodiazepines regularly ($\overset{¯}{x}$ = 2.031) were more likely to perceive risks than those who used them sporadically ($\overset{¯}{x}$ = 1.929) or never used them ($\overset{¯}{x}$ = 1.760). However, the model indicates that this group had a lower perception of psychosocial risks, i.e., 0.079 times lower (*β* = −0.059; *p* < 0.001).

Finally, although the use of antidepressants was not statistically significant (*β* = −0.063; *p*=0.006), a similar trend is observed: professionals who used them regularly ($\overset{¯}{x}$ = 2.095) reported a higher perception of psychosocial risks than those who used them sporadically ($\overset{¯}{x}$ = 1.958) or never used them ($\overset{¯}{x}$ = 1.772). Even so, the model suggests that, on average, nurses who used these drugs had a 0.063 times lower perception of psychosocial risks than those who did not use them.

## 4. Discussion

Psychosocial risks in the field of nursing remain a fundamental concern for both occupational health and quality of care [5]. The results obtained in this study, based on the ISTAS_Enfermería questionnaire, show the presence of significant risks which, while they may generally be considered moderate, are intensified by certain sociodemographic and health factors. These findings are in line with the previous literature highlighting the vulnerability of this group to demanding working conditions, high emotional burden and constant exposure to stressful situations [27].

In the specific context of Spain, institutional programmes promoted by the Ministry of Health should be mentioned. These programmes aim to enhance the mental and psychosocial well-being of healthcare professionals. The Mental Health Action Plan 2022–2024 (Ministry of Health, 2022) is one such example, as it includes specific measures to address psychological distress in the healthcare workplace. These measures include actions to prevent burnout, strengthen emotional support and promote healthy working environments [28].

Further analysis of the specific dimensions of psychosocial risk revealed significant associations with sociodemographic variables. Age showed weak but significant negative correlations with most risk dimensions, particularly with compensations (*ρ* = −0.34; *p* < 0.001) and social support (*ρ* = −0.10; *p* < 0.001), suggesting that younger professionals may perceive lower levels of institutional support and reward. In contrast, no significant relationship was found between age and double presence. Regarding civil status and education level, Kruskal–Wallis tests revealed statistically significant differences across groups in all dimensions except active work (*p*=0.064). The most pronounced differences were observed in compensations (*H* = 110.85; *p* < 0.001) and double presence (*H* = 96.18; *p* < 0.001), indicating that family and educational background may influence how professionals perceive workload balance and reward structures. These findings underscore the relevance of disaggregating psychosocial risks by subdimension and demographic profile in order to better tailor occupational health strategies.

One of the main findings of the present study is the inverse relationship between age and perceived psychosocial risk, as evidenced by a significant yet weak negative correlation. This pattern was consistent across the age distribution, which included a balanced representation of younger (< 30), mid-career (30–49) and older (≥ 50) nurses. This pattern has already been identified in the previous research, which suggests that younger professionals tend to have fewer resources to cope with work-related stress, thus facing higher levels of anxiety and emotional exhaustion [27]. Reduced work experience, together with possible job instability in early career stages, may explain this increased susceptibility [30]. However, it is important to consider the possibility that these associations could be influenced by occupational factors not included in the model. Such factors may include contract type, workload or work shift, which may be more unfavourable for younger professionals or those in the early stages of their careers. In addition, the association between age and perceived psychosocial risks was statistically significant; however, the effect size was weak. This also suggests a limited influence in practical terms and indicates that other contextual variables which were not measured (e.g., seniority, work setting or role stability) may moderate this relationship. These results reflect the independent associations of each variable with perceived psychosocial risk, while controlling for the influence of the other variables included in the model.

Regarding marital status, lower psychosocial risk scores were identified in married people, suggesting that the emotional and social support provided by a partner could function as a protective factor, as has already been reported in other studies [31]. This result reinforces the importance of considering the personal environment of the worker as a variable that can modulate the perception of work stress. However, the cohabitation situation may also reflect different stages of life or family responsibilities, which could interact with the work environment and alter the perception of risk.

A particularly relevant finding is the difference observed according to the level of education. While professionals with a doctorate or lower university studies had lower scores, those with a Master's degree showed higher levels of perception of psychosocial risks. This can be interpreted from different perspectives. On the one hand, it is possible that those with Master's degrees hold positions with greater responsibilities, which could increase the perceived demands of the job. On the other hand, these professionals may possess heightened critical awareness or specialised knowledge in the field of occupational risk prevention, which could amplify their sensitivity to adverse working conditions beyond the scope of objective exposure. It is recommended that subsequent studies give due consideration to these potential intervening variables in order to facilitate a more comprehensive understanding of the mechanisms underpinning these associations.

As regards health variables, the data revealed a strong association between the presence of chronic illnesses and the perception of psychosocial risks, confirming findings from studies linking work-related stress with a greater deterioration of physical and mental health [32]. This relationship can be understood in both directions: Impaired health may increase vulnerability to work-related stress, and constant exposure to psychosocial risks may aggravate pre-existing chronic conditions [33]. In addition, these findings are also aligned with national reports from the National Institute for Occupational Safety and Health (INSST, for its Spanish acronym) [34], which document similar risk patterns in the Spanish healthcare sector. Internationally, studies using the COPSOQ model in countries such as Denmark and Germany have reported comparable psychosocial profiles among nurses [35]. These similarities suggest a structural component in the psychosocial risk burden across healthcare systems, underscoring the need for systemic prevention strategies [36].

Self-perceived health was also found to exert a significant influence: respondents who rated their health status negatively had significantly higher scores on the ISTAS_Enfermería. This result is in line with the research which argues that subjective health perception is a sensitive indicator of general distress and the impact of the work environment on health [37]. In this sense, self-perceived health may function as an early risk marker that allows early preventive intervention mechanisms to be implemented [38].

One of the most consistent factors was the level of perceived stress, which showed a positive and significant correlation with psychosocial risks. This finding reinforces the key role of stress as a cross-cutting variable that interacts with multiple dimensions of both the work and the personal environment [39]. Indeed, accumulating evidence indicates that high levels of stress are associated with an increased likelihood of experiencing emotional exhaustion, interpersonal conflict and absenteeism [40].

The analysis of substance use offers relevant implications. In this study, the regular consumption of benzodiazepines or antidepressants is not interpreted as a psychosocial risk factor in itself, but rather as a possible consequence or coping mechanism in response to adverse work environments [41]. From this standpoint, consumption is conceptualised as a variable associated with exposure to psychosocial risks, rather than as a direct cause of their perception. However, the model shows that these groups had a lower perception of psychosocial risks compared to nonusers, which may be due to the regulatory effect of these substances on anxiety and depressive symptoms, leading to reduced sensitivity to occupational stressors [42]. This paradox between higher mean score and lower statistical association in the model may reflect compensatory mechanisms mediated by pharmacotherapy. Conversely, the fact that no significant differences were observed in relation to alcohol, tobacco or other substance use may be due to cultural factors and possible under-reporting of responses [43]. This discrepancy between bivariate and multivariate analyses may be explained by the existence of mediating or moderating effects, where the use of psychotropic medication could function as a coping mechanism that temporarily reduces the perception of occupational stressors. From a theoretical perspective, pharmacological strategies might dampen the emotional response to workplace demands, thereby attenuating subjective risk appraisal even in the presence of objectively adverse conditions. This is consistent with the cognitive appraisal theory of stress [44], which suggests that individual responses to stress depend not only on environmental demands but also on perceived coping resources. In this context, medication use could act as a psychological buffer, reducing the intensity of risk perception without necessarily improving the work environment. Further studies should explore these potential mediation or moderation pathways using structural equation modelling or longitudinal designs to better understand the dynamic interplay between coping strategies and psychosocial risk perception.

The number of sick leaves in the last year also showed a statistically significant relationship, but in the opposite direction to that expected: Those who had had two or more sick leaves showed a lower perception of psychosocial risks in the multivariate model. This finding can be interpreted from an adaptive perspective, in which sick leave functions as a psychological escape mechanism from distress, thereby facilitating a temporary disconnection from the stressful environment and consequently reducing the perception of risk upon return to work [45]. An alternative interpretation is that, following a critical phase that results in sick leave, individuals develop coping mechanisms or receive external interventions, such as institutional support or pharmacological treatment that influence their subsequent perception [46]. In this case, regular use of psychotropic drugs could contribute to emotional or cognitive regulation that mitigates the subjective assessment of the work environment, thus modulating the perception of risk without necessarily causing an objective change in working conditions [47]. Such mechanisms could be moderating or mediating the relationship between exposure to stressors and subjective perception of risk. Consequently, a diminished perception of risk does not inherently signify an enhancement in the work environment; rather, it constitutes a regulatory mechanism—adaptive or otherwise—that influences its evaluation.

Overall, the results indicate that the perception of psychosocial risks is the product of a complex interaction between individual factors (age, health and use of psychotropic drugs) and the work environment (level of responsibility, control and organisational support). This pattern is consistent with the demand–control–support (DCS) model, which posits that environments characterised by high demands, limited control over one's work and inadequate social support can lead to increased work stress and an elevated risk of burnout, particularly in caring professions such as nursing [48]. This correlational study of 1063 Japanese nurses found that high job demands and low control, as well as limited support from supervisors, were significantly associated with both the intention to leave the job and depressive symptoms.

The findings of this study have implications for both the theoretical understanding of psychosocial risks and their practical application in the management of healthcare staff. From an ethical perspective, the utilisation of this information should be guided by the principle of promoting support interventions for professionals who may be at risk. Such interventions may include mentoring programmes, reviews of working conditions or priority access to mental health resources. These measures are intended to foster an inclusive environment and should not be employed in a manner that could lead to the exclusion of these professionals from promotions or assignments. Accordingly, healthcare institutions bear the responsibility to employ these data in accordance with ethical criteria founded upon the principles of justice and nondiscrimination and to ensure the confidentiality of personal data. Moreover, collective well-being ought to be prioritised over individual performance.

In the field of healthcare, it is crucial to identify the specific factors associated with a higher perception of psychosocial risks. This is essential for designing more targeted and effective preventive strategies that focus on the well-being of nursing professionals. The findings of this study indicate that perceived stress emerges as a particularly influential factor, demonstrating a strong positive correlation with the perception of psychosocial risk (Tau-b = 0.318; *p* < 0.001). This finding indicates that interventions should prioritise the development of robust stress management programmes and the provision of accessible psychological support. Such programmes should focus on the development of resilience, the identification of coping strategies and the promotion of access to mental health services within the context of occupational health.

Furthermore, the significant association with the presence of chronic diseases ($\overset{¯}{x}$ = 1.841) and poor self-perceived health ($\overset{¯}{x}$ = 2.292 for poor health; $\overset{¯}{x}$ = 2.064 for fair health) suggests the need for integrated health surveillance programmes. These programmes should not only monitor physical health but also actively address the interaction between physical well-being and psychological distress, thereby facilitating early interventions for nursing professionals at risk.

Regarding the use of psychoactive substances, the findings of this study reveal that regular users of benzodiazepines ($\overset{¯}{x}$ = 2.031) and antidepressants ($\overset{¯}{x}$ = 2.095) reported higher mean scores in the perception of psychosocial risks. However, the multivariate model indicated a lower perception of psychosocial risks in these groups (*β* = −0.059 for benzodiazepines), which, as discussed, could reflect a compensatory or regulatory effect of pharmacotherapy. This complex finding suggests that interventions should not focus solely on substance use as a primary risk factor, but rather on identifying and addressing the underlying psychosocial stressors that may lead to the use of these substances as a coping mechanism. This underscores the importance of establishing confidential support systems and access to mental health resources within the workplace, with the aim of addressing the underlying causes of distress rather than merely the symptoms. A balanced approach is essential for ensuring that resources are allocated to combat the most impactful determinants, while also offering support for related coping behaviours.

Finally, although the number of sick leaves in the last year showed a statistically significant relationship in the bivariate analyses ($\overset{¯}{x}$ = 2.027 for two or more sick leaves), the multivariate model revealed a negative association (*β* = −0.135; *p* < 0.001). This finding suggests that sick leave could function as an adaptive coping mechanism, providing temporary relief from stressful environments and thus temporarily reducing the perception of risk upon return to work. Therefore, management strategies should not only focus on reducing absenteeism but also on understanding the underlying reasons for sick leave, such as unaddressed psychosocial stressors, in order to implement meaningful changes in the work environment to prevent the accumulation of distress.

Despite the significant results obtained, the explanatory power of the multivariate model remains limited (*R*^2^ = 29.6%), suggesting that important factors influencing the perception of psychosocial risks were not captured. Future research should incorporate key organisational and occupational variables such as type of employment (permanent vs. temporary), work shift patterns (e.g., night shifts and rotating shifts), workload and management style, as these have been consistently associated with job strain and psychological distress in nursing contexts. The inclusion of theoretical frameworks such as the job demands–resources (JD–R) model [49] or the ERI model [50] may also enhance explanatory depth. These frameworks allow for a more comprehensive understanding of how demands, control, support and perceived fairness in reward structures interact to shape workers' perceptions of stress and risk. Integrating such models could help clarify the complex interplay between individual and organisational determinants of psychosocial risk perception in healthcare environments.

### 4.1. Limitations

This study has some limitations to consider when interpreting the results. Firstly, the statistical model used managed to explain only a part of the variability in the perception of psychosocial risks (22.4%), which indicates that there may be other contributing factors that were not included in the analysis and that also influence this perception. This shows the complexity of the issue and suggests that it is a reality influenced by multiple elements.

These excluded variables encompass key occupational factors such as years of work experience, type of contract, work shift or workload, which have the potential to act as confounding variables in the observed associations, particularly in relation to age, educational level or marital status. The absence of these indicators limits the ability to fully interpret the mechanisms underlying the results obtained.

Additionally, as this was a cross-sectional study, the data were collected at a single point in time, which does not allow establishing cause and effect relationships between the variables analysed. For example, even if a relationship between stress, health status or medication use and risk perception was observed, it is not possible to be certain about which occurs first or how the different factors influence each other. The use of a nonprobabilistic snowball sampling strategy constitutes a major limitation of the study. This approach may have introduced selection bias by favouring the recruitment of participants with similar sociodemographic or professional characteristics, limiting sample diversity. Additionally, the lack of control over the recruitment chain restricts reproducibility and generalisability of the findings to the wider nursing population. Although the instrument included eligibility screening and data were reviewed for internal consistency, no IP filtering or direct tracking measures were implemented, which represents a further risk to sample integrity. These issues are consistent with the previous research noting the methodological constraints of snowball sampling in health studies [51].

The questionnaire was also voluntary and self-administered, which may have led to a participation bias. Respondents who perceived themselves to be more affected by their employment situation may have been more motivated to respond, which could have influenced the results and the representativeness of the sample. One important limitation of this study lies in the use of nonprobabilistic snowball sampling, which may introduce selection bias and reduce the national representativeness of the sample. Although participation was obtained from nurses working in diverse healthcare settings and across all autonomous regions of Spain, the absence of random sampling procedures limits the generalisability of the findings. Future studies should aim to use stratified or probabilistic sampling methods and consider disaggregating the data by autonomous region or institutional setting to allow a more detailed assessment of regional or organisational variability.

Although the number of participants was high and they came from different regions of Spain, there was a large proportion of women (87.3%), which partly reflects the reality of the nursing sector. However, this may limit the generalisability of the findings to other work settings or to groups of different compositions.

Due to the remarkably low number of participants reporting regular use of substances such as heroin or amphetamines, results related to these variables should be interpreted with caution. No inferential analyses were conducted for these subgroups due to insufficient statistical power.

Finally, questions on sensitive issues, such as substance use, may have been subject to some respondents' vague answers, either due to forgetfulness or to difficulty in talking openly about these issues. This may have affected the reliability of the data obtained.

### 4.2. Future Research and Perspectives

To address these limitations, future research should use follow-up designs over time. Such research should be complemented by qualitative methods and employ more comprehensive models that include organisational and psychosocial factors not addressed in this study.

The results of this study provide relevant evidence for different levels of intervention within the healthcare setting. Thus, this study facilitates action at the care and managerial levels and in the educational context.

At the care level, by identifying the factors associated with a higher perception of psychosocial risks, more effective preventive strategies can be designed, focussing on the well-being of nursing professionals. For example, findings on the impact of work-related stress, self-perceived health and the use of psychotropic drugs suggest the need to implement psychological support and stress management programmes for nurses.

At the management level, the data obtained can contribute to better human resources planning. When designing shifts, workloads or occupational health programmes, the sociodemographic characteristics of the staff (age, marital status, level of education and history of sick leave) should be incorporated into the planning. Also, evidence on the relationship between the level of education and the perception of risks highlights the importance of promoting ongoing professional development. This is so not only from an academic perspective, but this professional growth can also be a tool for providing professionals with coping and resilience skills.

In the educational context, the results point to the need to integrate content related to stress management, self-care and psychosocial factors in a more comprehensive manner in the undergraduate and postgraduate nursing curricula. In this way, future professionals would be better prepared to face the challenges inherent to their daily practice when they join the labour market.

## 5. Conclusion

The results of this study reveal that the perception of psychosocial risks among nurses in Spain is influenced by a combination of personal, professional and health-related factors. Variables such as age, marital status, educational level, self-perceived health status, work stress and psychotropic drug use showed a significant association with higher levels of psychosocial risk.

All these findings highlight the need for a holistic approach to the prevention and management of psychosocial risks that addresses both the conditions of the work environment and the individual characteristics of professionals. Similarly, emphasis is placed on the importance of promoting institutional policies aimed at the emotional well-being of nursing professionals through psychological support programmes, ongoing training and organisational strategies adapted to the specific needs of the nursing staff.

Despite the limitations of the explanatory model, the study data provide a solid basis for future research and for the design of more specific interventions in the healthcare, organisational and educational spheres. By addressing these factors, not only will the health of the nursing staff be improved but also the quality of care provided to the population.

## Figures and Tables

**Table 1:** Sociodemographic variables.

Variables	Total sample (*n* = 2765)	ISTAS21 mean score ($\overset{¯}{x}$/SD)	Contrast of hypotheses
Sex			
Male	341 (12.3%)	1.792 (SD = 0.562)	*p*=0.503^a^
Female	2414 (87.3%)	1.779 (SD = 0.494)	
Nonbinary	10 (0.4%)	1.874 (SD = 0.503)	
Age			
Mean	40.87 years	1.78 (SD = 0.5)	Tau b^b^ = −0.12 (*p* < 0.001)
Marital status			
Married	1186 (42.9%)	1.722 (SD = 0.494)	0.001^a^
Divorced	117 (4.2%)	1.751 (SD = 0.500)	
In a couple (cohabiting)	748 (27.1%)	1.840 (SD = 0.517)	
In a couple (not cohabiting)	242 (8.8%)	1.828 (SD = 0.484)	
Separated	41 (1.5%)	1.824 (SD = 0.561)	
Single	422 (15.3%)	1.819 (SD = 0.492)	
Widowed	9 (0.3%)	1.814 (SD = 0.546)	
Studies			
Bachelor's degree	588 (21.3%)	1.809 (SD = 0.480)	0.007^a^
Upper University studies (former educational model)	42 (1.5%)	1.820 (SD = 0.550)	
Lower University studies (former educational model)	704 (25.5%)	1.722 (SD = 0.464)	
Doctorate	59 (2.1%)	1.714 (SD = 0.641)	
Specialty	397 (14.4%)	1.751 (SD = 0.532)	
Master's degree	664 (24%)	1.831 (SD = 0.522)	
Expertise	298 (10.8%)	1.803 (SD = 0.504)	
Vocational training	13 (0.5%)	1.764 (SD = 0.493)	

*Note:* $\overset{¯}{x}$ Mean score.

Abbreviation: SD, standard deviation.

^a^Kruskal–Wallis H.

^b^Kendall's Tau B.

**Table 2:** Work-related variables.

Variables	Total sample (*n* = 2765)	ISTAS21 mean score ($\overset{¯}{x}$/SD)	Contrast of hypotheses
Chronic illness			
No	1972 (71.3%)	1.757 (SD = 0.497)	0.001^c^
Yes	793 (28.7%)	1.841 (SD = 0.513)	
Health status			
Poor	13 (0.5%)	2.292 (SD = 0.543)	0.001^a^
Fair	276 (10%)	2.064 (SD = 0.511)	
Good	1265 (45.8%)	1.797 (SD = 0.471)	
Very good	1027 (37.1%)	1.706 (SD = 0.491)	
Excellent	184 (6.7%)	1.632 (SD = 0.575)	
Level of stress			
Mean	6.86	1.781 (SD = 0.503)	Tau b^b^ = 0.318 (*p* 0.001)
Sick leave			
I have had NO sick leave in the last year	1444 (52.3%)	1.689 (SD = 0.494)	0.001^a^
I have had ONE sick leave in the last year	1028 (37.2%)	1.842 (SD = 0.484)	
I have had TWO OR MORE sick leaves in the last year	280 (10.1%)	2.027 (SD = 0.507)	
Prolonged/long-term sick leave (more than 1 year)	9 (0.3%)	1.837 (SD = 0.433)	
On leave of absence	2 (0.1%)	1.766 (SD = 0.141)	
Type of sick leave			
Sick leave due to accident at work	46 (3.9%)	1.891 (SD = 0.551)	0.991^a^
Sick leave due to occupational disease	102 (8.6%)	1.883 (SD = 0.481)	
Parental leave	97 (8.2%)	1.854 (SD = 0.483)	
Sick leave due to common illness	940 (79.3%)	1.895 (SD = 0.496)	
Frequency of alcohol consumption			
Never	672 (24.3%)	1.826 (SD = 0.532)	0.054^a^
Sporadically	1969 (71.2%)	1.767 (SD = 0.490)	
Regularly	124 (4.5%)	1.769 (SD = 0.526)	
Frequency of tobacco use			
Never	2225 (80.5%)	1.774 (SD = 0.505)	0.243^a^
Sporadically	238 (8.6%)	1.820 (SD = 0.476)	
Regularly	302 (10.9%)	1.802 (SD = 0.507)	
Frequency of cannabis use			
Never	2695 (97.5%)	1.779 (SD = 0.504)	0.062^a^
Sporadically	60 (2.2%)	1.833 (SD = 0.454)	
Regularly	10 (0.4%)	2.066 (SD = 0.309)	
Frequency of heroin use			
Never	2758 (99.7%)	1.781 (SD = 0.503)	0.566^a^
Sporadically	5 (0.2%)	1.573 (SD = 0.314)	
Regularly	2 (0.1%)	1.833 (SD = 0.047)	
Frequency of amphetamines use			
Never	2749 (99.4%)	1.780 (SD = 0.502)	0.343^a^
Sporadically	14 (0.5%)	2.009 (SD = 0.617)	
Regularly	2 (0.1%)	1.900 (SD = 0.471)	
Frequency of benzodiazepine use			
Never	2426 (87.7%)	1.760 (SD = 0.498)	0.001^a^
Sporadically	322 (11.6%)	1.929 (SD = 0.512)	
Regularly	17 (0.6%)	2.031 (SD = 0.597)	
Frequency of antidepressant use			
Never	2653 (95.9%)	1.772 (SD = 0.498)	0.001^a^
Sporadically	73 (2.6%)	1.958 (SD = 0.527)	
Regularly	39 (1.4%)	2.095 (SD = 0.611)	

*Note:* $\overset{¯}{x}$ Mean score.

Abbreviation: SD, standard deviation.

^a^Kruskal–Wallis H.

^b^Kendall's Tau B.

^c^Mann–Whitney.

**Table 3:** Model fit and significance of regression analysis.

Variable	Coefficient (*β*)	Degrees of freedom	F-statistic	*p* value
Marital status	0.072	2	14.316	< 0.001
Studies	0.104	2	30.811	< 0.001
Chronic illness	0.021	2	1.045	0.352
Health status	0.149	1	6.991	0.008
Sick leave	−0.135	1	39.622	< 0.001
Frequency of benzodiazepine use	−0.079	2	14.945	< 0.001
Frequency of antidepressant use	−0.063	2	7.476	0.06

*Note:R* ^2^ = 0.296; Fisher's *F* = 24,037; *p* < 0.001.

## Data Availability

All data are available under reasonable request to the corresponding author.

## Supporting Information

Supporting Table 1: Dimensions and specific items of the 20-item SUSESO/ISTAS21 short-version questionnaire in Spanish and English.

Description of Supporting Table 1: Supporting Table 1 presents the 20-item SUSESO/ISTAS21 short-version questionnaire in Spanish and English. It covers five dimensions: psychological demands, active labour and skills development, social support and leadership quality, compensation and double presence. Each item is scored on a five-point Likert scale from “*Always*” to “*Never*.”
