# Supplementary material for: Exploring Psychosocial Risk Factors Among Spanish Nurses: Links to Health and Professional Variables
Source: J Nurs Manag. 2025 Aug 26;2025:5531311. doi: 10.1155/jonm/5531311 (PMC12404832; doi:10.1155/jonm/5531311)
Supplement: Supporting Information — Additional supporting information can be found online in the Supporting Information section. [file 5531311.f1.docx]

Supplementary Table 1. Dimensions and specific items of the 20-item SUSESO/ISTAS21 short version questionnaire in Spanish and English.

| **Dimensión Exigencias Psicológicas** | **Dimension of Psychological demands at work** | **Siempre (Always)** | **La mayoría de las veces (Most of the time)** | **Algunas veces (Sometimes)** | **Sólo pocas veces (Rarely)** | **Nunca (Never)** |
| --- | --- | --- | --- | --- | --- | --- |
| 1 ¿Puede hacer su trabajo con tranquilidad y tenerlo al día? | 1.Can you do your job with peace of mind and keep it up to date? | 4 | 3 | 2 | 1 | 0 |
| 2. En su trabajo, ¿tiene usted que tomar decisiones difíciles? | 2. Do you have to make difficult decisions at work? | 4 | 3 | 2 | 1 | 0 |
| 3. En general, ¿considera usted que su trabajo le produce desgaste emocional? | 3. In general, do you consider that your work causes you emotional exhaustion? | 4 | 3 | 2 | 1 | 0 |
| 4. En su trabajo, ¿tiene usted que guardar sus emociones y no expresarlas? | 4. In your work, do you have to keep your emotions to yourself and not express them? | 4 | 3 | 2 | 1 | 0 |
| 5. ¿Su trabajo requiere atención constante? | 5. Does your job require constant attention? | 4 | 3 | 2 | 1 | 0 |
| **Dimensión Trabajo Activo y Desarrollo De Habilidades** | **Dimension of Active Labour and Skills Development** | **Siempre (Always)** | **La mayoría de las veces (Most of the time)** | **Algunas veces (Sometimes)** | **Sólo pocas veces (Rarely)** | **Nunca (Never)** |
| 6. ¿Tiene influencia sobre la cantidad de trabajo que se le asigna? | 6. Do you have influence over the amount of work assigned to you? | 4 | 3 | 2 | 1 | 0 |
| 7. ¿Puede dejar su trabajo un momento para conversar con un compañero/a? | 7. Can you leave your job for a minute to talk to a colleague? | 4 | 3 | 2 | 1 | 0 |
| 8. ¿Su trabajo permite que aprenda cosas nuevas? | 8. Does your job allow you to learn new things? | 4 | 3 | 2 | 1 | 0 |
| 9. Las tareas que hace, ¿le parecen importantes? | 9. Do the tasks you do seem important to you? | 4 | 3 | 2 | 1 | 0 |
| 10. ¿Siente que su empresa o institución tiene una gran importancia para usted? | 10. Do you feel that your company or institution is of great importance to you? | 4 | 3 | 2 | 1 | 0 |
|  |  |  |  |  |  |  |
| **Dimensión Apoyo Social y Calidad de Liderazgo** | **Dimension of Social Support and Leadership Quality** | **Siempre (Always)** | **La mayoría de las veces (Most of the time)** | **Algunas veces (Sometimes)** | **Sólo pocas veces (Rarely)** | **Nunca (Never)** |
| 11. ¿Sabe exactamente qué tareas son de su responsabilidad? | 11. Do you know exactly what tasks are your responsibility? | 4 | 3 | 2 | 1 | 0 |
| 12. ¿Tiene que hacer tareas que usted cree que deberían hacerse de otra manera? | 12. Do you have to do tasks that you think should be done differently? | 4 | 3 | 2 | 1 | 0 |
| 13. ¿Recibe ayuda y apoyo de su superior inmediato? | 13. Do you receive help and support from your immediate superior? | 4 | 3 | 2 | 1 | 0 |
| 14. Entre compañeros y compañeras, ¿se ayudan en el trabajo? | 14. Do you help each other at work? | 4 | 3 | 2 | 1 | 0 |
| 15. Sus jefes inmediatos, ¿resuelven bien los conflictos? | 15. Do your immediate bosses resolve conflicts well? | 4 | 3 | 2 | 1 | 0 |
| **Dimensión Compensaciones** | **Dimension of Compensation** | **Siempre (Always)** | **La mayoría de las veces (Most of the time)** | **Algunas veces (Sometimes)** | **Sólo pocas veces (Rarely)** | **Nunca (Never)** |
| 16. ¿Está preocupado/a por si le despiden o no le renuevan el contrato? | 16. Are you worried about whether you will be fired or have your contract renewed? | 4 | 3 | 2 | 1 | 0 |
| 17. ¿Está preocupado/a por si le cambian de tareas contra su voluntad? | 17. Are you worried about having your tasks changed against your will? | 4 | 3 | 2 | 1 | 0 |
| 18. Mis superiores me dan el reconocimiento que merezco | 18. My superiors give me the recognition I deserve | 4 | 3 | 2 | 1 | 0 |
|  |  |  |  |  |  |  |
| **Dimensión Doble Presencia** | **Dimension of Double Presence** | **Siempre (Always)** | **La mayoría de las veces (Most of the time)** | **Algunas veces (Sometimes)** | **Sólo pocas veces (Rarely)** | **Nunca (Never)** |
| 19. Cuándo está en el trabajo, ¿piensa en las exigencias domésticas y familiares? | 19. When you're at work, do you think about domestic and family demands? | 4 | 3 | 2 | 1 | 0 |
| 20. ¿Hay situaciones en las que debería estar en el trabajo y en la casa a la vez? (para cuidar un hijo enfermo, por accidente de algún familiar, por el cuidado de abuelos, etc.) | 20. Are there situations where you should be at work and at home at the same time? (to take care of a sick child, due to an accident of a relative, to take care of grandparents, etc.) | 4 | 3 | 2 | 1 | 0 |
